# Computer-aided Image Processing of Angiogenic Histological

**DOI:** 10.4021/jocmr2009.12.1274

**Published:** 2009-12-28

**Authors:** Matvey Sprindzuk, Alexander Dmitruk, Vassili Kovalev, Armen Bogush, Alexander Tuzikov, Victor Liakhovski, Mikhail Fridman

**Affiliations:** a United Institute of Informatics Problems, National Academy of Sciences of Belarus, Belarus; b Minsk City Hospital for Oncology, Belarus

## Abstract

This article reviews the questions regarding the image evaluation of angiogeneic histological samples, particularly the ovarian epithelial cancer. Review is focused on the principles of image analysis in the field of histology and pathology. The definition, classification, pathogenesis and angiogenesis regulation in the ovaries are also briefly discussed. It is hoped that the complex image analysis together with the patient’s clinical parameters will allow an acquiring of a clear pathogenic picture of the disease, extension of the differential diagnosis and become a useful tool for the evaluation of drug effects. The challenge of the assessment of angiogenesis activity is the heterogeneity of several objects: parameters derived from patient’s anamnesis as well as of pathology samples. The other unresolved problems are the subjectivity of the region of interest selection and performance of the whole slide scanning.

**Keywords:**

Angiogenesis; Image processing; Microvessel density; Cancer; Pathology

## Introduction: angiogenesis definition and its role in ovarian function and pathology

The aim of this paper is to provide the reader with a brief understandable description of angiogenesis research facts and concepts, especially the histology sample image analysis strategies and its problems.

Angiogenesis, the development of the new capillaries from the already existing vessels, is an important process of the normal physiologic activities such as the cyclic ovarian function, wound healing and embryonic growth. Another definition: Angiogenesisis is the stimulation of the new endothelial cell growth and the new blood vessel development [1]. Angiogenesis was first described by Hunter in 1787 [2]. Despite the enormous amount of information concerning angiogenesis, to the best of our knowledge, there are only few literature sources describing the image analysis of angiogenesis [3-24] and even less about this topic in the field of ovarian cancer research [25-27].

**Figure 1:**
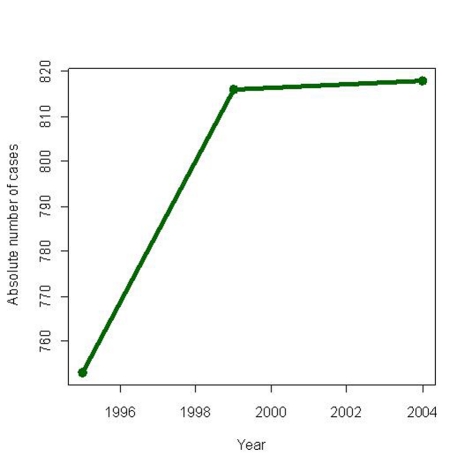
Graphical description of ovarian cancer epidemiology (case incidence) in Belarus, 1995, 1999, 2004. Data taken from reference [34].

Ovarian cancer is one of the major causes of female oncologic death worldwide (Fig. 1). It causes more than 140,000 deaths annually in women worldwide. About 21,650 cases of invasive ovarian cancer resulting in 15,520 deaths were predicted to occur in 2008 [28]. For decades, the classical treatment of this disease has been the platinum chemotherapy and surgery.

All ovarian tumors are divided into the following categories [29]: (1) Surface derived (serous, mucinous, endometrioid and Brenner tumor); (2) Germ cell tumors (cystic teratoma, dysgerminoma, yolk sac tumor); (3) Sex-Cord derived (thecoma-fibroma, granulosa-thecal cell tumor, Sertoli-Leydig cell and gonadoblastoma); (4) Neoplasias metastatic to ovary: Krukenberg tumor.

Current concepts regarding the origins and molecular pathology of ovarian cancer suggest that the dysfunction of K-ras, b-raf, BRCA1, p53 genes and several others often occur in these patients. Among the predisposing diseases are endometriosis, initially benign cysts and cystadenomas [30].

By establishing a correlation between angiogenesis and cancer development and progression we can arrive at a more complete understanding regarding angiogenesis, cancer and ultimately individual recurrence and survival [31].

Increased vascularity and angiogenesis occur in support of actively proliferating tumor cells and thus blood vessel parameters may have a potential application as diagnostic and prognostic indicators [32].

**Figure 2:**
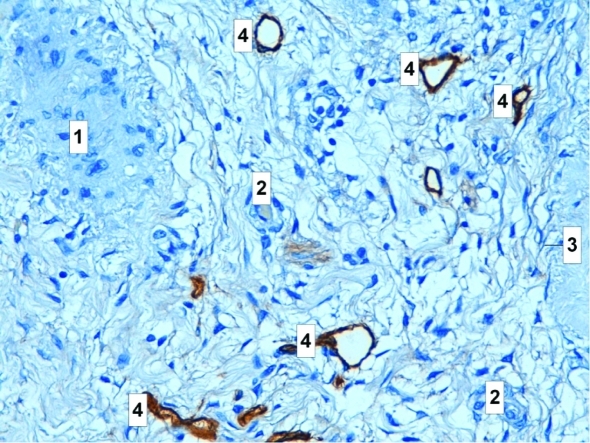
Histology sample of epithelial ovarian cancer, lymphatic microvessel staining D2-40, podoplanin antibody, peroxidase and hematoxylline, magnification x200. 1, atretic follicle; 2, blood vessel; 3, corpus albicans; 4, stained lymphatic vessels.

That is our opinion that angiogenesis activity is especially dynamic and unstable in the ovaries and it is probably almost impossible to estimate all the microenvironment effects influencing the angiogenesis expansion in any organ or tissue system. What we can see on the histological image are only the tubular structures (blood and lymphatic capillaries) and the surrounding cells within a connective tissue matrix (Fig. 2).

Angiogenesis is a key aspect of normal cyclical ovarian function. Follicular growth and the development of the corpus luteum (CL) are dependent on the proliferation of new capillary vessels. The process of selection of a dominant follicle in monovular species has been also associated with angiogenesis, as there is evidence that selected follicles possess more elaborate microvascular networks than other follicles. After blood vessel growth, the blood vessels regress, suggesting the coordinated action of inducers as well as inhibitors of angiogenesis in the course of the ovarian cycle [33].

Apparently, angiogenesis can be investigated on the level of the entire organism, as on the levels of an organ system, a separate organ, a tissue and cells. Angiogenesis on the histology sample section is assessed by the eye of the trained pathologist (manually), using the graticule, via Chalkey stereologic method and applying the image processing software.

## Angiogenesis classification

Two main types of angiogenesis have been described: sprouting, the expansive growth of the vascular network; and remodeling, the rebuild of the vessel net. It is considered that the first type predominates in malignant tumor growth. However, Fox and colleagues (2007) do not include the remodeling in classification of the angiogenesis. They have suggested the classification of tumor neovascularization with 5 types. (1) Angiogenesis: the generation of new blood vessels from the existing vasculature; (2) Vasculogenesis, the de novo generation of blood vessels from endothelial cell progenitors, as occurs in the embryo; (3) Vascular remodeling: intussusceptive vascular growth, referring to vascular network formation by insertion of interstitial tissue columns into the vascular lumen and subsequent growth of these columns resulting in partitioning of the vessel lumen, endothelial cell division is not required for this form of vascular remodeling [35]; (4) Glomeruloid angiogenesis, which refers to the highly complex vascular aggregates that resemble glomeruli of the kidney, composed of a network of capillaries that are variably lined by basement membrane and pericytes; (5) Vascular mimicry defined as a complete capillary network composed of tumor cells themselves rather than vascular endothelial cells that conduct blood [36].

## Vascular endothelial growth factor (VEGF)

VEGF/VPF (now termed VEGF-A) was first identified in 1993 by Senger and colleagues in ascites fluid of tumors in rodents. Years later, VEGF was found in the malignant effusions of several human tumors. VEGF is a heparin binding glycoprotein that occurs in at least four molecular isoforms as the result of alternative VEGF mRNA splicing [1].

The VEGF family is an increasingly important set of factors in ovarian cancer. VEGF has been established experimentally as one of the major inducers of ascites and its expression can be at least in part a consequence of a positive feedback loop where the ovarian cancer cells produce LPA, which in turn induces VEGF, causing ascites with high circulating concentrations of LPA. Several studies demonstrated the importance of other members of the VEGF family, including VEGF-C and VEGF-D, which induce de novo formation of lymphatic capillaries (lymphangiogenesis). These factors have also been shown to promote metastasis via the lymphatics. Further, the short isoform of the alternatively spliced VEGF receptor-3, the receptor tyrosine kinase receptor for VEGFs-C and -D, is related to development of lymph node metastasis in breast cancer. VEGF-C is induced by hypoxia, making it also likely to be involved in the development of ovarian cancer or the ascites of ovarian cancer. The role of the angiopoietins and the other VEGF family members has not yet been examined in detail in ovarian cancer [37].

VEGF receptors family is not the only molecules expressed differently in tumor and normal endothelial cells, and angiogenesis is not the only way of the cancer expansion, however its predominant pathogenic role is clearly established. The mentioned molecular group includes: (1) VEGF receptors; (2) Integrins (Avb3, Avb5); (3) Adhesion molecules (E-selectin, Endoglin); (4) Glycoproteins (Prostate-specific antigen); (5) Proteases.

Three main approaches to targeting angiogenesis in the treatment of ovarian cancer have been described. The first has been to target VEGF itself, the second to block the VEGF binding site on its cell surface receptors, and the third to inhibit tyrosine kinase activation and downstream signaling with small molecules at the intracellular level [28].

The extensive description of ovarian cancer pathology and treatment is beyond the scope of the article.

## Major markers of the blood vessels

The most commonly used antibodies to highlight tumor blood vessels are those against Factor VIII related antigen, CD31/PECAM-1, and CD34. Factor VIII related antigen is a part of the von Willebrand factor complex and plays a role in the coagulation cascade. The platelet endothelial cell adhesion molecule CD31/PECAM-1 is a transmembrane glycoprotein involved in cell adhesion. CD34 is a surface glycoprotein of unknown function [1]. The both known blood vessel endothelial and lymphatic (panendothelial) markers are CD31, CD34, CD105, cadherin (adhesion molecules), VEGF2, VEGF3, Tie-2/Tek (tyrosine kinase receptors), CCL20/MIP-3-alpha (CC-chemokine), and E9 (endothelial protein) [2,38,39], (Table 1).

**Table 1:** Panendothelial Angiogenesis Markers

Endothelial marker	Laboratory and clinical qualities
CD31	- commonly used
	- cross-reacts with plasma cells
	- frequent antigen loss occurs due to fixatives containing acetic acid
	- infiltrates sometimes obscure microvessels, especially single cell sprouts
CD34	- the most reproducible endothelial cell highlighter
	- highlight perivascular stromal cells
CD105	- proliferation-associated and hypoxia-inducible protein
	- preferentially expressed in the activated endothelial cells participating in neoangiogenesis
	- undetectable or weakly expressed in vessels of normal tissues
Tie-2/Tek	- endothelium-specific receptor tyrosine kinase
	- identifies stromal vessels
E-9	- protein specific for activated/proliferating endothelial cells

Text partially derived from [2, 38]

### Angiogenesis regulation

The process of angiogenesis is regulated by the cytokines, growth factors, the interaction between the endothelial cells and components of the extracellular matrix and the surrounding cells, including macrophages, smooth muscle cells, fibroblasts. The initiation, further development and fading of the angiogenesis activity are dependent on the balance of the angiogenic and antiangiogenic factors in the endothelial cell microenvironment. Angiogenesis can be represented as the consequence of stages. For the malignant tumor it is a process starting from the single dormant endothelial cell which can grow to the magnificent vascular network providing the multi faceted homeostasis. The critical moment of the latent endothelial cell transformation to an active dividing cell is called the angiogenic switch. Ovarian function is dependent on the establishment and continual remodeling of a complex vascular system. This enables the follicle and/or CL to receive the required supply of nutrients, oxygen and hormonal support as well as facilitating the release of steroids. Moreover, the inhibition of angiogenesis results in the attenuation of follicular growth, disruption of ovulation and drastic effects on the development and function of the CL. It appears that the production and action of vascular endothelial growth factor A (VEGFA) is necessary to all these stages of development. The extensive vascularization of the CL enables it to receive one of the highest blood flows per unit tissue mass. Luteal blood flow remains at pre-ovulation levels in the collapsed follicle, but thereafter gradually increases in parallel with increases in luteal volume and coincides with increases in progesterone. The controlled, physiological angiogenesis that accompanies folliculogenesis, ovulation and luteal development requires the coordinated activity of multiple cell types and different angiogenic factors. It appears that VEGFA regulates angiogenesis by stimulating endothelial proliferation, migration and survival and is required at all stages from a secondary follicle right through to the mature CL. However, the often overlooked FGF2 plays a more dynamic role and is likely to be critical during the follicle- luteal transition [40].

The same factors that control angiogenesis in other organs also control angiogenesis in the endometrium. It is believed that VEGF by interacting with its receptor VEGFR-1 and VEGFR-2 plays a major role in controlling endometrial angiogenesis. However, there is no clear cyclic pattern of VEGF or VEGF receptor expression as it for example occurs in the ovary. Many of the other angiogenesis-regulating cytokines have also been detected in the endometrium including the positive angiogenesis regulators bFGF, TGF-β, TNF-α, and IL-8 and the negative regulator thrombospondin [41].

Interestingly, the three investigated gene polymorphisms did not correlate with any of the investigated clinico-pathological parameters. In univariate and multivariate models, only FIGO stage and patient's age at diagnosis, but not any polymorphism or any haplotype, were correlated with patients' overall survival. In this large multi-center study, the investigated VEGF gene polymorphisms were not associated with prognosis in patients with ovarian cancer [42].

In the normal cycling ovary of the mare the most intensive expression of the angiogenic factors VEGF A, VEGF B, Ang1, Ang2 and their receptors VEGF-R1, VEGF-R2 and Tie2 can be detected in granulosa, lutein and theca interna cells as well as in thecal vessels during the periovulatory period. In this context, especially VEGF A, Ang2, VEGF-R2 and Tie2 seem to be important for angiogenesis during follicular and luteal development, while Ang1 serves for vessel stabilization. VEGF B and VEGF-R1 are probably only of secondary importance. In contrast, during luteal regression and follicular atresia, the ?ndings in luteal regression and follicular atresia show that, in the absence of VEGF A, factor Ang2 and its receptor Tie2 contribute substantially to vessel regression [43].

## Angiogenesis staging

This includes: (1) existing vessel endothelial cell basal membrane degradation and fragmentation influenced by the action of metalloproteases; (2) endothelial cell migration to the stromal tissue and extracellular matrix proteolytic degradation; (3) endothelial cell proliferation, new capillary tube formation, fusion of the formed vessels and its network development; (4) endothelial cell proliferation and migration suppression and fate under the influence of the angiogenic factors [44-47].

## The concepts of the automated cancer diagnosis and the assessment of angiogenesis activity using image analysis for histological samples

Computers help health care professionals make robust decisions in various spheres of medicine, and in the realm of pathology image processing the opportunities of the software packages are of a special value. A perfect program should do anything what a human can and at the same time requires a minimum user intervention and conditions and performs as fast as possible. Unfortunately, not all the phenomena we can observe and investigate could be transformed in digits and this is the dilemma of the qualitative and quantitative analysis in research. The limitation of the image analysis software designed for the processing of angiogenesis samples is the problem of estimating the hypervascular areas which could be relatively easily assessed by an experienced pathologist. Computer can tell the scientist (this is the-output) what is the area of pixels had ascribed to the structures defined by the programmer who had put the computer language code (such as Java, C++ or C#). More complex features of an image such as the fractals could also be calculated by software. The obtained image features can be correlated with a spectrum of qualitative and quantitative parameters (patients age, comorbidity, biochemical values etc) and the diagnosis parameters are derived eventually. The fact that only few conducted studies have a weak statistic power, the meta-analysis of similar trials can serve as the reliable source of knowledge to make decisions about the right treatment and follow-up for patients.

Good software can calculate the relationship between the target objects on the screen and the areas occupying the recognized structures. Pathologists can not estimate and compute the exact numerical values looking at the image, but they can choose the region of interest for the further computations. The ideal software developed to evaluate angiogenesis should choose the necessary regions of interest on the image slide, to recognize correctly all available structures, but these are the unresolved questions of modern medical image analysis software engineering.

The first method of the quantitative evaluation of angiogenesis has been proposed and developed by Brem, the target of his research were the brain tumors [48].

At the beginning of research activities, the measure of the angiogenesis process activity was the vessel area, stained by a coloring reactive or via the immunochemical method. The search for the new reliable biomarkers of angiogenesis is ongoing, and along with this branch of investigation the new approaches to the angiogenesis image have been suggested. Since Weidner and colleagues estimated the microvessel density in the most vascularized area (hot spot) in their pioneering work in 1991, the same technique with slight modifications has been used widely to assess the prognostic value of angiogenesis in various types of carcinomas. Afterwards, the Chalkey method based on Chalkey eyepiece graticule was introduced to provide a quicker and more objective procedure for measuring tumor vascularity. Currently, the Chalkey assay with CD34 immunostaining has been suggested as a standard method for angiogenesis quantification in solid tumor sections in an international consensus report, although the basis for the consensus has been questioned by others.

Microvessel density assessment is the most commonly used technique to quantify intratumoral angiogenesis. It uses panendothelial immunohistochemical staining of blood microvessels, mainly with Factor VIII antigen (F. VIII Ag or von Willebrands factor), PECAM/CD31, or CD34; rarely with integrin v3, CD105, or type IV collagen. The first step in Weidners approach is the identification by light microscopy of the area of highest neovessel density, the so called "hot spot", by scanning the whole tumoral section at low power. Then, individual microvessels are counted at a higher power (x200 field) in an adequate area (e.g., 0.74 mm^2^ per field using x20 objective lens and x10 ocular). Any stained endothelial cell or clusters separate from adjacent vessels are calculated as a single microvessel, even in the absence of vessel lumen. Each single count is expressed as the highest number of microvessels identified at the hot spot. Some authors use Chalkey count or computerized image analysis systems, both aimed to minimize the subjectivity in the quantification of MVD. The Chalkey count consists of applying a 25-point eyepiece graticule on several hot spots (usually 3). The graticule is oriented to allow the maximum number of points to hit on or within the areas of stained microvessel profiles (Chalkey grid area: 0.196 mm^2^) [49]. Quantitating angiogenesis by the Chalkey method represents a relative area estimate of the vessels rather than a true vessel count. This has been thought to be an advantage by improving the objectivity of evaluation because the method abolishes one of the highly observer-dependent steps in microvessel density measuring: the decision whether two immunostained and adjacent structures were the reflection of one single or two separate blood vessels. Supporting this assumption, the Chalkey method has been shown to have less observer variation than estimation of microvessel density in breast cancer. An important observer-dependent step still remains in the selection of the densely vascularized areas, “vascular hot spots” for microvessel quantitation. However, the reproducibility is not necessarily optimized by choosing the same hot spot area. It was shown that high angiogenesis measured by the Chalkey method predicts poor overall survival in the whole study group and in the advanced stage ovarian cancers. Researchers suggested that, in order to define the clinical significance of this finding in more detail, further studies on other patient materials and perhaps comparison with other evaluation methods of angiogenesis in ovarian cancer should be conducted [50].

Steps of automated cancer diagnosis include: (1) Preprocessing (noise elimination and segmentation); (2) Feature extraction (feature types determination and selection); (3) Diagnosis (development of learning algorithms and statistical tests performance) [2].

In order to obtain the data on microvessel features, digital slide undergoes several stages of the image processing procedures: (1) Color deconvolution; (2) Light and dark staining thresholding; (3) Connecting endothelial cells in regions; (4) Completing vessels procedure; (5) Microvessel analysis [51].

The features of the histopathology sample image are classified according to their nature into the following groups: (1) morphological (vessel shape, perimeter); (2) texture (edge smoothness, roughness, coarseness etc.); (3) fractal (fractal dimension and lacunarity); (4) topological (spatial arrangement); (5) intensity based (color brightness) [52].

The morphological approach quantifies the size and shape of a cell or its nucleus. The textural approach makes use of spatial inter-relationships for the pixels to extract features and quantifies properties such as the smoothness, regularity, and coarseness of the image. The intensity based approach employs the distribution of the intensity values of pixels to define its features. The topological approach quantifies the spatial distribution of the cells within a tissue. Although these approaches lead to promising results in automated cancer diagnosis, they suffer from one or both of the two problems: (1) the difficulty of determining the exact locations of cells/nuclei in the biopsy image or (2) the noise that arises from its staining process. The cell-graph approach relies on cluster formation in cancerous cells to define their distinctive features. In this method, we identify the cell clusters on a tissue image as the nodes and compute the spatial dependency between every pair of these nodes to probabilistically assign the edges. Unlike the previous demonstrations, the cell-graph approach makes use of the cell clusters instead of the individual cells. Therefore, it eliminates the necessity of determining the exact locations of cells/nuclei on a tissue image, which allows using the low-magnification images. Furthermore, this approach relies on the dependency between the cell clusters rather than the pixels themselves and does not directly use the pixel values in feature extraction. Because of that, it is likely immune to noise inherit in a biopsy image [53].

The term *fractal* (from Latin *fractus* - irregular, fragmented) applies to objects in space or fluctuations in time that possess a form of *self-similarity* and cannot be described within a single absolute scale of measurement. Fractals are recurrently irregular in space or time, with themes repeated like the layers of an onion at different levels or scales. Fragments of a fractal object or sequence are exact or statistical copies of the whole and can be made to match the whole by shifting and stretching. Sequential fractal scaling relationships are observed in many physiological processes. Spatial structures of many living systems are fractal. Fractal geometry has evoked a fundamentally new view of how both nonliving and living systems result from the coalescence of spontaneous self-similar fluctuations over many orders of time and how systems are organized into complex recursively nested patterns over multiple levels of space. The fractal models may be used for image segmentation, texture classification, shape from-texture, and the estimation of 3-D roughness from image data. Related algorithms and suitable procedures are already implemented in some image processing software. If the parameters gained by the analysis are taken to supply classification problems where textural information is to be processed, the structures under consideration do not necessarily have to be fractals. In cellular morphometry cells and nuclei can be quantitatively described by measuring their fractal dimension [54].

Fractal dimension is a measure of how complicated a self-similar figure is. In a rough sense, it measures how many points lie in a given set. Somehow, though, fractal dimension captures the notion of ‘how large a set is’. Fractal object has a property that more fine structure is revealed as the object is magnified, similarly like morphological complexity means that more fine structure (increased resolution and detail) is revealed with increasing magnification. Fractal dimension measures the rate of addition of structural detail with increasing magnification, scale or resolution. The fractal dimension, therefore, serves as a quantifier of complexity. Fractals that have the same fractal dimension may look very different, they have different ‘texture’, more specifically, different lacunarity. Lacunarity is a counterpart to the fractal dimension that describes the texture of a fractal. It is strongly related with the size distribution of the holes on the fractal and with its deviation from translational invariance. Roughly speaking, if a fractal has large gaps or holes it has high lacunarity; on the other hand, if a fractal is almost translationally invariant it has low lacunarity. Lacunarity (from the Latin lacuna for lack, gap or hole) measures structural variation or inhomogenities that may be manifested by texture. In a restrictive sense it is a measure of the lack of rotational or translational invariance. In a more general sense, lacunarity is a measure of non uniformity (heterogeneity) of structure or the degree of structural variance within an object. Lacunarity is usually defined in terms of mass related distribution [54].

Syntactic structure analysis (SSA), a method often used for quantification of tissue architecture (the arrangement of vascular spatial positions in case of angiogenesis). This approach uses a binary representation of the centers of gravity of individual vessels to construct graphs or diagrams of which the characteristics are applied as contextual parameters. Examples of such figures are the Voronoi diagram, the Gabriels graph and the minimum spanning tree, which may give insight into the division of the blood supply inside the tumor and the immediate and distant vascular neighborhood relationships, respectively [55].

The fractal and syntactic analysis recently showed its reliability for the purposes of the research field of angiogenesis micropathology. Several authors have described a broad spectrum of descriptors for this relatively novel method [55-57]. These descriptors can provide a multi faceted comprehensive picture of the angiogenesis microscopic assay.

Entropy, correlation and image contrast are an image characteristics derived via the texture analysis. The color analysis, pixel color comparison, histology image texture analysis all have been applied in attempts to find an appropriate robust and reliable approach to the assessment of angiogenesis net [58,59].

Color is an important factor in defining structures in biological science. The fields of histology and pathology are founded on the use of special dyes and staining procedures that label cells or structures of interest with a defining color. Examples include the use of special stains for specific biochemical groups, e.g., trichrome stain for matrix and cell tissue, and the use of chromogenic substrates in enzyme linked-immunochemistry, e.g., immunoperoxidase labeling, and reporter enzyme constructs in transfected cells, e.g., b-galactosidase-transfected cells. Hence color image analysis (CIA), in which colored objects of interest are isolated or segmented from surrounding structures for subsequent measurement, is becoming an increasingly important tool in modern pathology and cell biology [60].

The methodology for MVD practical assessment is rather simple. It is similar to the original Weidner’s approach described above. The tumor is scanned at low power (x40-100) (center), and the three areas that contain the highest number of discrete microvessels are selected. The three hot spot areas containing the maximum number of discrete microvessels should be identified by scanning the entire tumor at low power (x40 and x100). This is the most subjective step of the procedure. It has been demonstrated that the experience of the observer determines the success of identifying the relevant hot spots. Poor selection will in turn lead to an inability to classify patients into different prognostic groups. Observers spend time in a laboratory where a period of training can be undertaken. Ideally, comparisons between hot spots chosen by an experienced investigator and trainee should be performed and continued on different series until there is more than 90% agreement. Training can be completed by assessing sections from a series that already contain prognostic information. Inexperienced observers tend to be drawn to areas with dilated vascular channels, often within the sclerotic body of the tumor. These central areas together with necrotic tumor should be ignored. Vascular lumina or erythrocytes are not a requirement to be considered a countable vessel; indeed, many of the microvessels have a collapsed configuration. Although the hot spot areas can occur anywhere within the tumor, they are generally at the tumor periphery, making it important to include the normal tumor interface in the representative area to be assessed. Vessels outside the tumor margin by one x200-250 field diameter and immediately adjacent to benign tissues should not be counted. The procedure takes 2-5 min. Once selected, a 25-point Chalkey point eyepiece graticule at x200-250 should then be oriented over each hot spot region so that the maximum number of graticule points are on or within areas of highlighted vessels. Particular care should be taken in the occasional case (<1% breast cancers) for which an intense plasma cell infiltrate can mimic a hot spot and obscure the underlying tumor vasculature. Plasma cells can otherwise be disregarded on morphological grounds. The mean of the three Chalkey counts is then generated for each tumor and used for statistical analysis. The procedure takes 2-3 min. For the intratumoral microvessel density index, any endothelial cell or endothelial cell cluster separate from adjacent microvessel, tumor cells, or matrix elements is considered a countable vessel. Those that appear to be derived from the same vessel if distinct should also be counted. Again, vessel lumens and erythrocytes are not included in the criteria defining a microvessel. There is no cutoff for vessel caliber. The procedure takes 3-6 min [61].

Angiogenesis measured by MVD method correlates with the tumor behavior. There is a vast body of facts showing that the angiogenesis intensity and a higher MVD are associated with the metastases development, poor prognosis and life longevity shortage in breast [36,62-64], urine bladder [65,66], renal cell [67] and stomach cancer patients [68-72]. In the area of ovarian cancer clinical investigations and histology sample image analysis and pathology differential diagnosis, the data on the prognostic significance and reliability of MVD is controversial [1,73-77]. Bamberger and Perret in 2002 concluded that in contrast with the breast tumor evaluation, there is no clear correlation of the MVD parameter with an ovarian cancer patient age, tumor stage, growth rate, dimension, ascites; for the various histological subtypes of ovarian cancer, MVD is different [1]. The further research in this direction elucidated the similar contradictions [74]. It can be hypothesized that the discrepancy of various conclusions is explained by the heterogeneity of patients genotypes in context of the biomarker expression. Other reasons are the differences of micro sample images. Several scientists have discussed the disadvantages and limitations of the MVD evaluation techniques [78-80]. Brown based on his personal observational experience and the literature data of other research groups postulated that this method is not an appropriate one for the precise angiogenesis activity assessment in large scale randomized clinical trials [80].

Microvascular density would be a good indicator of therapeutic efficacy, but it has not been as useful for efficacy as it has been for prognosis. Since the early studies, hundreds of reports have examined the prognostic value of microvascular density in several forms of cancer. Nevertheless, despite the initial confirmatory publications, numerous reports appeared in the literature that failed to show a positive association between increasing tumor vascularity and reduced patient outcome, and caution as to the clinical utility of tumor angiogenesis is being urged. However, many of these negative studies may result from significant differences in methodologies [2].

Quantification of tumor angiogenesis by counting microvessels in immunostained tissue sections was ranked by The College of American Pathologists in category III, encompassing “all factors which are not sufficiently studied to demonstrate their prognostic value”. The issues of methodological variation mentioned include: antibody selection, type of ?xative used, methods of counting vessels, calculation of microvessel density, observer variability (especially of the selection of the field in which to count) and cut-off value for ‘increased’ vascularity [35]. Thus, the negative qualities of the ‘gold standard’ are the reasons for the investigation in order to find a new better approach to the stained capillary specimen image analysis.

The alternative ways of the quantitative angiogenesis evaluation are the determination of microvessel density in the randomly selected areas or in an intentionally chosen such as the tumor tissue edges. Whether the both methods provide the same biologic information or one is better in terms of some features over another, this question is open for the debates. Besides the MVD density itself, there are several parameters which can be determined and quantified analyzing the image of the angiogenesis network: mean microvessel surface proportions, vascular area fraction, absolute number of vessels, absolute microvessel number and their perimeter, vessel angle, length and squares of the defined capillary categories [10,81,82]. The object of the angiogenesis measurement of the Angio quant program (software which is freely available for the aims of scientific research at www.cs.tut.fi) is the network of the joint tubular complexes. The software can determine the length and the size of these tubular complexes and the number of network connections (branch points) in a complex. The developers concluded that the distribution of the length parameter in evaluation of the experimental angiogenesis obeys the power law [16,83]. Moreover, in his research papers, Blacher et al mentioned the vessel length density, in particular, radially arranged, capillary fractal dimension and the mean capillary diameter as the evaluation characteristics [84].

When the MVD is calculated, the separate object stained for example with CD34 biomarker is registered as 1 vessel. It is assumed that by its nature, the immunochemical vessel staining is not an ideal diagnostic technique, because its important drawback is the fact that the surrounding vessels or the cluster of them can be recognized as 1 object, which will seriously negatively bias the future conclusions of the research [85,86].

Another interesting research field is the image densitometry applying the Feulgen reaction, which have been widely used for the DNA analysis [87-95]. The VEGF mRNA may be an appropriate target of this method for the indirect evaluation of the angiogenesis growth and intensity. In its initial formulation, the histochemical reaction developed by Robert Feulgen was used simply for the detection of DNA in the nucleus (Feulgen and Rossenbeck 1924), but since the demonstration that it is both specific and stoichiometric for DNA it has become the most important means of staining nuclear DNA for densitometric quantification. The protocol has been modified frequently and substantially since its early development, but the basic components have not been altered [96].

Spectral analysis has been applied for the image processing derived via microscopy for several years but to our knowledge this modality did not gain the wide spread use for the angiogenesis activities investigations [97,98]. Spectral methods are based on the calculation of the eigenovectors and eigenovalues of a matrix derived from the affinities of the pixels in the image [99]. According to Laitakari (Finland), integrated optical density (IOD) is defined as the sum of individual pixel staining intensity values of the objects, e.g. nuclei or vessels. This is referred to later as total staining intensity (TSI). Average staining intensity (ASI) is defined as TSI divided by the number of pixels in an object, thus being independent of object size. Accuracy, maximum 0.1μm, is defined as the smallest measurable unit achieved repeatedly from the same measurement. Sensitivity is defined as the minimum level of light intensity needed to separate two different measurements, specificity is defined as the minimal measurable difference between different light emissions, reliability is the difference when the same specimen is measured similarly on two different occasions, and repeatability is the difference in measurements when the same measurement is repeated several times [31].

## Conclusions

The problem of angiogenesis in medicine touches such questions as the development and the implementation of the novel methods for a vascularized tissue staining, the elaboration of the reliable, precise and robust methods for the machine recognition and processing of the cell structures visualized on the organ tissue sections. The discovery of the new endothelial biomarkers has allowed developing immunochemical reactives for the clear and concise vascular structures visualization, and the quantitative computer-assisted analysis of this image provides a stable, non emotional unbiased assessment, which serves as the evidence-based fundament for the really scientific concepts and conclusions.

The principal problem of angiogenesis research is the heterogeneity of various aspects of the research spheres: the unique genetic package inherited to every patient creates the unique picture of angiogenesis network. The ovarian tumors are classified according to their tissue structure features and in order to conclude a scientifically and, in particular statistically true and valid statements, it is necessary to select a group of patients having similar characteristics. The limitations of many studies are the minor number of similar objects. These obvious rules are necessary to determine the validity of such conclusions as the role of microvessel density and the other features derived from the image analysis of a histology section.

Many pathologists prefer to use one representative section taken from the gross tumor specimen, and this section is scanned, then several shots are taken (usually 3) of the hot spot areas. To our opinion, for the research purposes, the entire slide should be scanned or if the microscope has no a function like this, the pathologist should take as many camera shots as possible to cover the significant surface of the sample at a various magnification rates. This method is appropriate when the researchers aim to investigate the image objects which are not recognizable for a human eye neither with nor without a microscope. The disadvantage of this approach is the problem that this task is excessively time-consuming, and the derived slides from the whole slide surface might lack its representativeness and eventually be a low-yield in terms of the useful scientific information.

## Abbreviations

Ang: angiopoetin
ASI: average staining intensity
CL: corpus luteum
DNA: deoxyribonucleic acid
FGF: fibroblast growth factor
FIGO: International Federation of Gynecology and Obstetric
LPA: lipopolysacharide antigen
MVD: microvessel density
PECAM: platelet endothelial cell adhesion molecule
PI3K: phosphatidylinositol-3-kinase
TSI: total staining intensity
VEGF: vascular endothelial growth factor
IL: interleukin
IOD: integrated optical density
TNF: tumor growth factor
Tie2: endothelium-specific receptor tyrosine kinase
3-D: three dimensional
